# Definitive Hosts of *Versteria* Tapeworms (Cestoda: Taeniidae) Causing Fatal Infection in North America

**DOI:** 10.3201/eid2204.151446

**Published:** 2016-04

**Authors:** Laura M. Lee, Roberta S. Wallace, Victoria L. Clyde, Annette Gendron-Fitzpatrick, Samuel D. Sibley, Margot Stuchin, Michael Lauck, David H. O’Connor, Minoru Nakao, Antti Lavikainen, Eric P. Hoberg, Tony L. Goldberg

**Affiliations:** University of Wisconsin–Madison, Madison, Wisconsin, USA (L.M. Lee, A. Gendron-Fitzpatrick, S.D. Sibley, M. Lauck, D.H. O’Connor);; Milwaukee County Zoo, Milwaukee, Wisconsin, USA (R.S. Wallace, V.L. Clyde, A. Gendron-Fitzpatrick);; Colorado State University, Fort Collins, Colorado, USA (M. Stuchin);; Wisconsin National Primate Research Center, Madison (M. Lauck, D.H. O’Connor, T.L. Goldberg);; Asahikawa Medical University, Asahikawa, Hokkaido, Japan (M. Nakao);; University of Helsinki, Helsinki, Finland (A. Lavikainen);; United States National Parasite Collection, Beltsville, Maryland, USA (E.P. Hoberg)

**Keywords:** Cestoda, Taeniidae, Versteria, metacestode, Mustelidae, ermine, Mustela ermine, mink, Neovison vison, North America, host, fatal infection, tapeworm, parasites

## Abstract

We previously reported fatal infection of a captive Bornean orangutan with metacestodes of a novel taeniid tapeworm, *Versteria* sp. New data implicate mustelids as definitive hosts of these tapeworms in North America. At least 2 parasite genetic lineages circulate in North America, representing separate introductions from Eurasia.

Taeniid tapeworms (Cestoda: Taeniidae) comprise 4 proposed genera: *Taenia*, *Echinococcus*, *Hydatigera*, and *Versteria* (*1*). Until recently, genetic data were absent for *Versteria* sp. in North America. However, in 2014, we reported an unusual case of fatal metacestode (larval stage of tapeworm) infection in a captive Bornean orangutan (*Pongo pygmaeus*); the causative agent was identified as a novel *Versteria* genotype (*2*).

As previously described (*2*), the orangutan was born at a zoo in Colorado, USA, and was rejected by his birth mother. Approximately 10 months later, he was transported to the Milwaukee County Zoo in Milwaukee, Wisconsin, USA, for adoption by a surrogate mother. At ≈5 years of age, he died unexpectedly from acute respiratory distress due to disseminated infection with an unknown agent. A combination of metagenomics and gene-specific DNA sequencing revealed the etiologic agent to be a previously unknown *Versteria* lineage in its larval form.

We obtained wild mustelids (carnivores of the family Mustelidae) from Colorado, near where the animal was born, and Wisconsin, near where the animal died. We targeted mustelids because they are definitive hosts of *V. mustelae* tapeworms in Europe (*3*) and *V. brachyacantha* tapeworms in Africa (*4*). Colorado samples were submissions to the Denver Museum of Nature and Science, and Wisconsin samples were obtained from a local fur trapper. We also examined mustelids from Oregon, USA, as part of an ongoing investigation of *Versteria* spp. tapeworms in the Nearctic region and Eurasia.

From 4 mustelids from Colorado (1 otter [*Lontra canadensis*], 2 ermine [*Mustela erminea*], and 1 mink [*Neovison vison*]), we recovered 1 adult tapeworm from a female ermine collected 178 km from where the orangutan was born. From 17 mustelids from Wisconsin (1 mink, 5 long-tailed weasels [*M. frenata*], and 11 ermine), we recovered 1 adult tapeworm from a male ermine collected 56 km from where the orangutan died. From 17 mustelids from Oregon (1 mink, 1 ermine, and 15 long-tailed weasels), we recovered 1 adult tapeworm from an adult mink of unknown sex. All tapeworm specimens were fragmented, lacking intact scolices, or both, which prevented complete morphologic description; however, microscope examination of mature segments from the Wisconsin and Oregon specimens revealed structures consistent with those of parasites in the genus *Versteria* (Figure 1).

**Figure 1:**
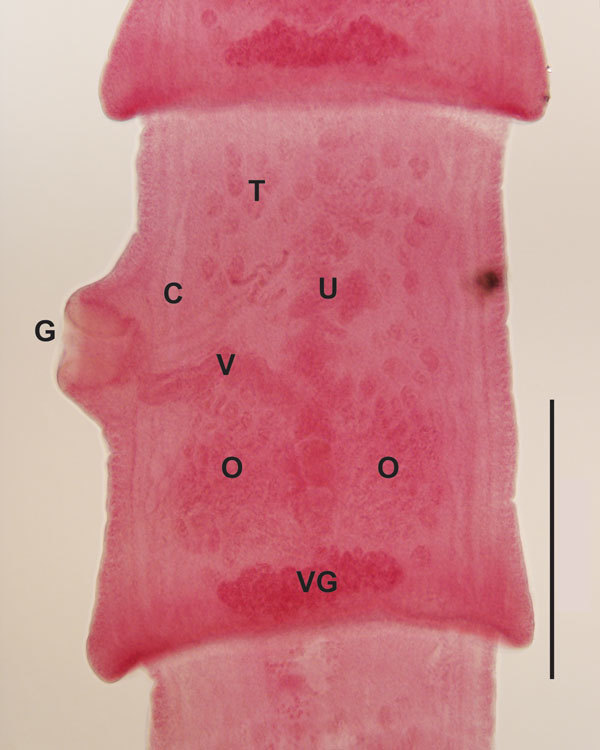
Microscope image of a mature segment of an adult *Versteria* sp. tapeworm recovered from an ermine in Wisconsin, USA (original magnification ×10). Characteristic reproductive structures are visible, including genital pore (G), cirrus sac (C), vagina (V), ovary (O), testes (T), uterine stem (U), and vitelline gland (VG). Tapeworm specimens were preserved in 70% ethanol for concurrent morphologic and molecular analyses. A series of proglottids was subsampled from each worm as a basis for sequencing; remaining strobila was stained, cleared and mounted in Canada balsam as permanent vouchers based on standard methods (*5*). Specimens are deposited in the Museum of Southwestern Biology, Parasitology Division, University of New Mexico, Albuquerque, New Mexico, USA (accession no. MSB 23169), and in the collections of the Denver Museum of Nature and Science, Denver, Colorado, USA (accession no. DZTM.3170). Scale bar indicates 500 µm.

We sequenced 396 bp of the mitochondrial cytochrome c oxidase subunit 1 (*cox1*) gene from the 3 new adult tapeworm specimens according to previously published methods (*2*). The sequences of the tapeworms from the Colorado ermine and the Oregon mink were 99.5% and 99.2% similar, respectively, to the sequence from the orangutan, placing these new specimens confidently within the same *Versteria* lineage (Figure 2). By contrast, the sequence from the tapeworm from the Wisconsin ermine was only 90.7% similar to the sequence from the orangutan, making it a heretofore unrecognized lineage that clusters more closely with parasites from Eurasia (Figure 2).

**Figure 2:**
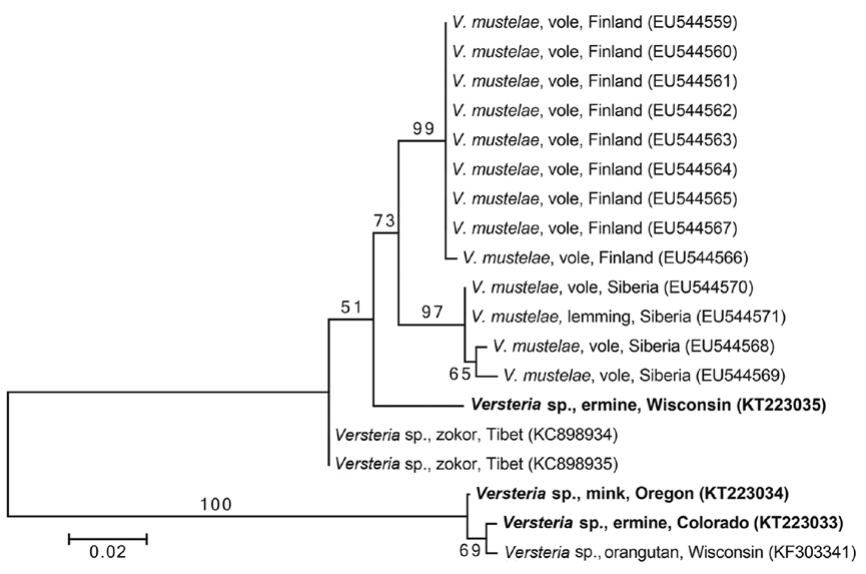
Phylogenetic tree of members of the genus *Versteria* (Cestoda: Taeniidae). The tree was constructed from a DNA sequence alignment of cytochrome c oxidase subunit 1 genes. The maximum-likelihood method was used, with the likeliest model of molecular evolution (Hasegawa-Kishino-Yano model with invariable positions), which was chosen by using MEGA6 (*6*). Numbers next to branches indicate bootstrap values (%), estimated from 1,000 resamplings of the data, and the tree is rooted at the midpoint of the longest branch. Taxon labels indicate parasite species, intermediate or definitive host, and geographic origin (GenBank accession nos. in parentheses). Bold indicates sequences from this study (from adult parasites and definitive hosts). Scale bar indicates nucleotide substitutions per site.

To investigate the hosts from which adult tapeworms were recovered, we amplified and sequenced 751 bp of the mustelid cytochrome b (*cytb*) gene, using DNA extracts from adult tapeworm material. To do this, we used previously published methods (*7*,^8^) with modified PCR primers MVZ45_must_F (5′-CAGTNATAGCAACAGCATTCATAGG-3′) and MVZ14_must_R (5′-GCTCTCCATTTTTGGTTTACAAGAC-3′). This effort was successful, demonstrating that adult tapeworm material contains sufficient host-associated DNA for such analyses. The resulting sequences indicated that the ermine from Colorado and Wisconsin (GenBank accession nos. KT223030 and KT223032, respectively) belonged to the continental clade, which is broadly distributed across the Nearctic region (*9*). The sequence for the Oregon mink (GenBank accession no. KT223031) closely matched published sequences for other mink from North America but was otherwise phylogeographically uninformative.

We next assessed whether parasites of the genus *Versteria* were present in wild carnivores on and near the grounds of the Milwaukee County Zoo. We conducted systematic searches of the zoo grounds (i.e., outside of enclosures) for carnivore feces. We also trapped carnivores, using live traps baited with fish, and then immediately released them, collecting any feces deposited in the trap, and we collected feces from mink from a local wildlife rehabilitation center. These efforts yielded 51 samples (3 from skunks [*Mephitis mephitis*], 3 from coyotes [*Canis latrans*], 6 from long-tailed weasels, 8 from mink, and 31 from raccoons [*Procyon lotor*]), which we examined by microscope, using standard flotation and sedimentation methods to identify parasite eggs (Table 1). We then used the ZR Fecal DNA MiniPrep kit (Zymo Research, Irvine, CA, USA) to extract DNA directly from feces samples and amplified and sequenced the *cox1* gene (*2*). Resulting sequences were unambiguous and revealed a diversity of parasites (not only taeniids, and in some cases representing putative novel lineages), but none matched *Versteria* spp. (Table 2).

**Table 1:** Parasites in wild carnivore feces samples collected on the grounds of and near the Milwaukee County Zoo, Milwaukee, Wisconsin, USA, 2014*

Parasite	Host, % positive				
	Coyote, n = 3	Long-tailed weasel, n = 6	Mink, n = 8	Raccoon, n = 31	Skunk, n = 3
Ascarid	–	–	–	12.9	–
*Baylisascaris procyonis*	–	–	–	35.5	–
Cestode	33.3	50.0	16.7	25.8	66.7
Coccidia	66.7	33.3	–	22.6	33.3
*Cystoisospora* spp.	–	–	–	3.2	–
*Giardia* spp.	–	–	16.7	25.8	–
Hookworm	–	–	–	12.9	33.3
Strongylid	–	16.7	33.3	12.9	33.3
*Trichuris* spp.	–	–	33.3	22.6	–
Other†	66.7	33.3	50.0	3.2	–

*–, not present. †Other parasites were unidentified metastrongyles, nematodes, and protozoans.

**Table 2:** Parasites identified by DNA sequencing of the *cox1* gene in samples of wild carnivore feces collected on the grounds of and near the Milwaukee County Zoo, Milwaukee, Wisconsin, USA, 2014*

Host	GenBank accession no.†	Most similar to	% Similarity‡
Mink	KT223036	*Alaria alata*	91.2 (HM022221)
Raccoon	KT223037	*Baylisascaris procyonis*	100.0 (KC172104)
Long-tailed weasel	KT223038	*Parafilaroides normani*	89.6 (KJ801815)
Skunk	KT223039	*Taenia crassiceps*	86.9 (EU544549)
Mink	KT223040	*Toxascaris leonina*	93.1 (JF780951)

*Only samples with parasite eggs resembling those of cestodes were tested; mink samples were from a local wildlife rehabilitation center. Sequences matching noncestode parasites indicate nonspecificity of PCR primers; results do not exclude the possibility of mixed infections. *cox1*, cytochrome c oxidase subunit 1. †For parasite sequences newly generated during this study. ‡Nucleotide similarity to the most similar sequence in GenBank (accession no. in parentheses) as of June 26, 2015.

Our results demonstrate that the genus *Versteria* is a species complex in North America. The new lineage identified in Wisconsin clusters with parasites formerly known only from Eurasia (Figure 2). Preliminary assessments suggest that this same lineage infects ermine as far away as the Northwest Territories (NWT) of Canada (E.P. Hoberg et al., unpub. data) and that the lineage responsible for fatal infection of an orangutan (*2*) also infects muskrats (*Ondatra zibethicus*) in Idaho, USA, and the NWT (A. Lavikainen et al., unpub. data). To the extent that current sampling reflects the distribution of *Versteria* lineages in North America, there appears to be a western lineage (represented by Colorado, Oregon, and Idaho, and the NWT) and a northern continental lineage (represented by Wisconsin and the NWT), with sympatry in the NWT. We note that this pattern parallels the phylogeography of ermine in North America, perhaps reflecting postglacial expansion of host and parasite after the Pleistocene (*9*). More *Versteria* lineages will likely be found, as evidenced by a novel lineage in zokor (*Eospalax baileyi*, a fossorial rodent) recently described from Tibet (*10*) (Figure 2). *Versteria* lineages have clearly entered the Nearctic region from the Palearctic region at least twice, probably reflecting Beringian biogeographic processes of faunal expansion that are important drivers of the evolution of mammals and the organisms parasitizing them (*11*).

Our findings shed light on the origins of the infection that proved fatal to the orangutan. We found an adult *Versteria* sp. tapeworm with a nearly identical DNA sequence in Colorado, where the orangutan was born; however, a *Versteria* sp. tapeworm from Wisconsin, where the orangutan died, was genetically divergent. Moreover, we found no evidence of *Versteria* tapeworms on the grounds of the Milwaukee County Zoo or nearby. Taeniid metacestodes encyst and can remain dormant for years before asexual multiplication (*12*). We therefore suspect that the orangutan became infected where it was born (Colorado) and carried the latent infection to where it died ≈4 years later (Wisconsin). This animal’s sudden progression to disease remains a mystery, perhaps indicating immune deficiency or another precipitating factor, consistent with reports of disseminated taeniid infection in other hosts (*13*).

In general, our findings underscore that exotic animals in zoo settings are susceptible to infections harbored by local wildlife and that transport of such animals can complicate inferences about the origins of these locally acquired infections. We reiterate that taeniid tapeworms of the genus *Versteria* should be considered a threat to captive apes (*2*), and we recommend that wild mustelids, such as ermine and mink, be excluded or removed from the grounds of zoos where apes have access to outdoor environments. Given the close relationship between apes and humans, we also suggest increased vigilance for zoonotic infections.
